# Glycyrrhizin ameliorating sterile inflammation induced by low-dose radiation exposure

**DOI:** 10.1038/s41598-021-97800-8

**Published:** 2021-09-15

**Authors:** Hyung Cheol Kim, Hyewon Oh, Je Sung You, Yong Eun Chung

**Affiliations:** 1 Department of Radiology, Yonsei University College of Medicine, 50-1 Yonsei-ro, Seodaemun-gu, Seoul, 03722 Republic of Korea; 2 Department of Emergency Medicine, Yonsei University College of Medicine, Seoul, Republic of Korea

**Keywords:** Translational research, Diagnostic markers

## Abstract

Glycyrrhizin (GL) is a direct inhibitor of HMGB1 which acts as an alarmin when excreted into the extracellular space. High-dose radiation in radiotherapy induces collateral damage to the normal tissue, which can be mitigated by GL inhibiting HMGB1. The purpose of this study was to assess changes in HMGB1 and pro-inflammatory cytokines and to evaluate the protective effect of GL after low-dose radiation exposure. BALB/c mice were irradiated with 0.1 Gy (n = 10) and 1 Gy (n = 10) with GL being administered to half of the mice (n = 5, respectively) before irradiation. Blood and spleen samples were harvested and assessed for oxidative stress, HMGB1, pro-inflammatory cytokines, and cell viability. HMGB1 and pro-inflammatory cytokines increased and cell viability decreased after irradiation in a dose-dependent manner. Oxidative stress also increased after irradiation, but did not differ between 0.1 Gy and 1 Gy. With the pretreatment of GL, oxidative stress, HMGB1, and all of the pro-inflammatory cytokines decreased while cell viability was preserved. Our findings indicate that even low-dose radiation can induce sterile inflammation by increasing serum HMGB1 and pro-inflammatory cytokines and that GL can ameliorate the sterile inflammatory process by inhibiting HMGB1 to preserve cell viability.

## Introduction

On a molecular level, radiation causes DNA damage either directly with high linear energy transfer or indirectly with low-dose radiation through the formation of free radicals^1^. However, the question remains on whether low-dose radiation actually harms humans because various DNA repair mechanisms do exist^2,3^. As a conservative approach is usually chosen for radiation protection, the linear no-threshold (LNT) model in which cancer incidence is considered to increase linearly as the amount of radiation exposure increases, regardless of how small the amount of radiation exposure is, continues to be adopted as the standard for clinical practice^4^. Recent large-scale studies support this model in truth because even low-dose radiation has been found to contribute to an excess incidence risk of cancer and death^5,6^. Therefore, there is a need to reduce the biological effects of even low-dose radiation exposure.

High mobility group box 1 (HMGB1) is a nuclear protein which regulates transcription and chromatin remodeling^7,8^. It acts as an important part of innate immunity and is actively secreted from immune cells by stimuli such as ischemic-reperfusion injury, trauma, or microbial infection and can also be passively released from necrotic cells into the extracellular space^9,10^. The discharged HMGB1 acts as a signal molecule for damage-related molecular patterns (DAMPs)^7,11^ and promotes inflammatory responses similar to pathogen-associated molecular patterns (PAMPs)^12^. In addition, HMGB1 stimulates monocytes to promote the synthesis of pro-inflammatory cytokines such as TNF-α, IL-1α, IL-1β, IL-6 and IL-8^13,14^, and combines to toll-like receptor (TLR)-2, TLR-4, or receptor for advanced glycation end products (RAGE) to mediate inflammatory reactions^15,16^. Ionizing radiation is a stimulus of sterile inflammation that induces the translocation of HMGB1 from the nucleus to the cytoplasm and extracellular space^17,18^.

Glycyrrhizin (GL) is a triterpenoid glycoside from the licorice root, Glycyrrhiza glabra. It directly binds to HMGB1 and inhibits its chemotactic and mitogenic activities^19^. According to previous studies, GL can ameliorate ischemic-reperfusion injury, hepatotoxicity, and acute kidney injury by inhibiting the HMGB1 pathway^20,21^. It can also mitigate radiation-induced tissue injuries such as radiation-induced acute lung injury^22^ or radiation enteritis^23^. However, past studies have only looked into HMGB1 and the abovementioned protective effects of GL after relatively high doses of radiation (20 Gy and 6.5 Gy, respectively)^22,23^. Hence, the purpose of this study was to assess changes in HMGB1 and pro-inflammatory cytokines and to investigate the protective effect of GL after low-dose radiation exposure.

## Results

### Mitigation of oxidative stress by glycyrrhizin

**Figure 1:**
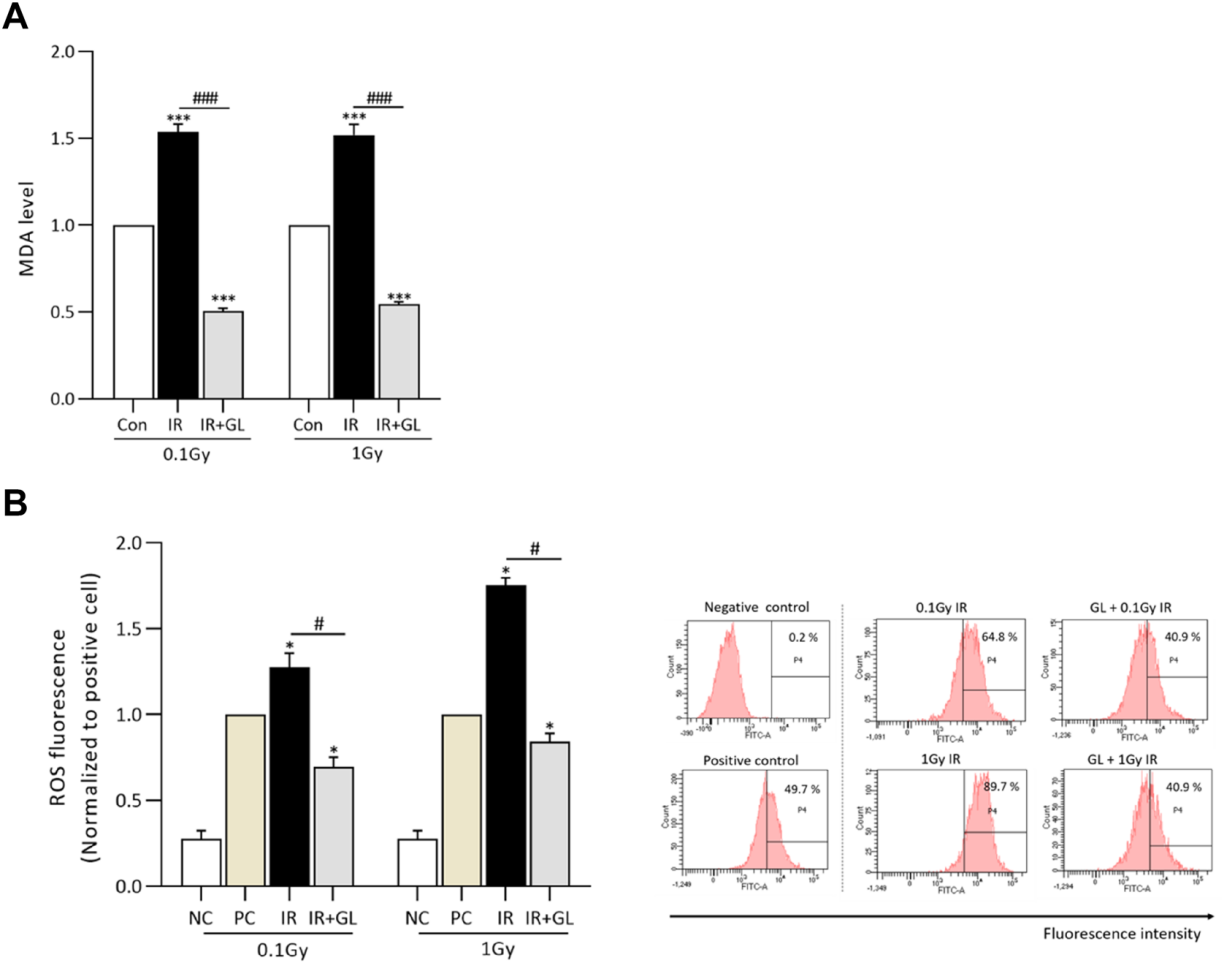
Oxidative stress measured by the MDA assay according to radiation dose. (**A**) The graph shows the normalized levels of MDA for the IR with/without GL groups compared to the control group. Oxidative stress was significantly higher in the IR only groups compared to the controls, whereas it was attenuated in the IR + GL groups. (**B**) ROS fluorescence was measured using flow cytometry at 24 h. It significantly higher in the IR only groups compared to the positive controls, whereas it was lower in the IR + GL groups. Measeured values normalized to the positive controls (= H_2_DCFDA stained cells). Results are expressed as means ± SEM from duplicate experiments (n = 5) performed in each group. The asterisk (*) indicates statistically significant differences between the control and IR groups (both IR only and IR + GL). The hash sign (#) indicates significant differences between the IR only groups and IR + GL groups. *p < 0.05, ***p < 0.001 #*P* < 0.05 and ###*p* < 0.001. Abbreviation: *IR* irradiation, *GL* glycyrrhizin, *MDA* malonaldehyde acid, *ROS* reactive oxidative species, *NC* negative control, *PC* positive control.

We observed how glycyrrhizin affects oxidative stress in the low-dose radiation exposure group. Oxidative stress was significantly higher in the IR only groups (both in the 0.1 Gy IR only and 1 Gy IR only groups) compared to the control group (*p* < 0.001, respectively), whereas oxidative stress in the IR + GL groups (0.1 Gy IR + GL and 1 Gy IR + GL groups) was significantly lower compared to the IR only groups (*p* < 0.001, respectively). There was no significant difference in oxidative stress between the 0.1 Gy IR only and 1 Gy IR only groups and none between the 0.1 Gy IR + GL and 1 Gy IR + GL groups (Fig. 1A). Also, we confirmed ROS fluorescence using FACS. The ROS levels were higher in the IR only groups (both the 0.1 Gy IR only and 1 Gy IR only groups) compared to the positive control group (*p* = 0.028, respectively), whereas the ROS levels in the IR + GL groups (0.1 Gy IR + GL and 1 Gy IR + GL groups) was lower compared to the IR only groups (*p* = 0.028, respectively) (Fig. 1B). In addition, we investigated DNA damage due to oxidative stress using 8-OHdG ELISA. Oxidative stress was significantly higher in the IR only groups (both the 0.1 Gy IR only and 1 Gy IR only groups) compared to the control group (*p* = 0.028, respectively), whereas it was significantly lower in the IR + GL groups compared to the IR only groups (*p* = 0.028, respectively) (Supplementary 1A). To confirm DNA damage, we additionally evaluated the expression of γ-H2A.X which is a DNA double strand breakdown (DSB) marker. The expression of γ-H2A.X was higher in the 1 Gy IR only group compared to the 0.1 Gy IR only group 30 min after radiation exposure. With the pretreatment of glycyrrhizin, DNA DSB was attenuated in both IR only groups. At 24 h after radiation exposure, DSB was not detected in both the 0.1 Gy IR only and 1 Gy IR only groups. (Supplementary 1B).

### Expression of HMGB1 and other pro-inflammatory cytokines

**Figure 2:**
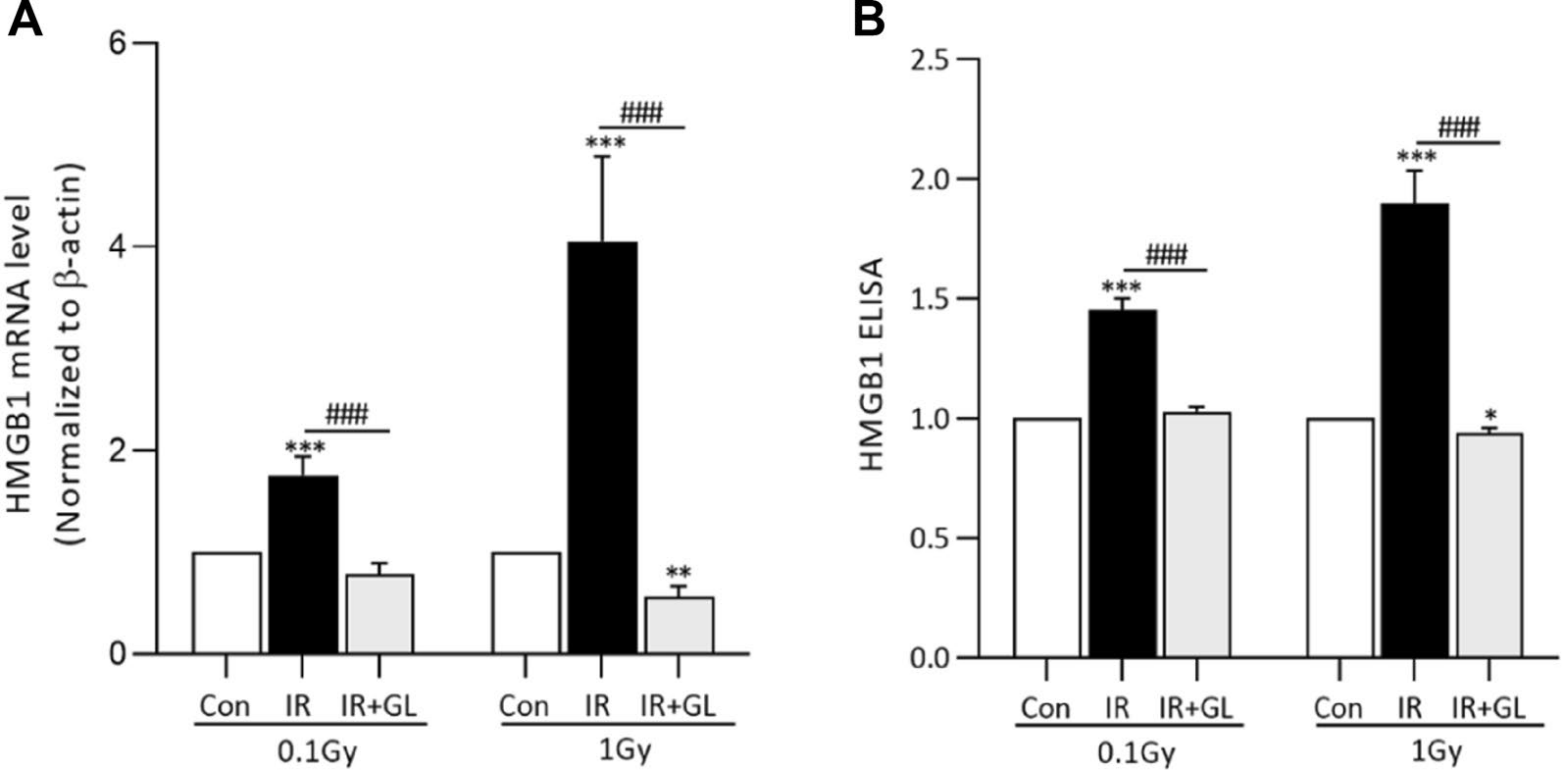
mRNA expression (**A**) and serum protein levels (**B**) of HMGB1 according to irradiation dose. Both the mRNA expression (**A**) and serum protein levels (**B**) of HMGB1 were significantly higher in the IR only groups (both in 0.1 Gy and 1 Gy) compared to the control group, whereas, they were significantly lower in the IR + GL groups compared to the IR only groups. Results are expressed as means ± SEM from duplicate experiments (n = 5) performed for each group. The asterisk (*) indicates statistically significant differences between the control and IR groups (both IR only and IR + GL). The hash sign (#) indicates significant differences between the IR only groups and IR + GL groups. ***p < 0.001, ###*p* < 0.001. *IR* irradiation, *GL* glycyrrhizin.

The mRNA expressions of HMGB1 and pro-inflammatory cytokines after radiation exposure were assessed to evaluate the mitigation effects of glycyrrhizin. The mRNA expression level of HMGB1 was significantly higher in the IR only groups compared with the control group (*p* < 0.001, both in 0.1 Gy and 1 Gy). It was significantly more elevated in the 1 Gy IR only group compared to the 0.1 Gy IR only group (1.76 times higher for the 0.1 Gy IR only group and 4.06 times higher for the 1 Gy IR only group compared to the control group, *p* = 0.023). In contrast, the mRNA expression of HMGB1 in the IR + GL group was significantly lower in the IR + GL group than the IR only groups, and this was true for both the 0.1 Gy and 1 Gy groups (Fig. 2A). Similarily, serum HMGB1 was significantly higher in the IR groups compared to the control group for both 0.1 Gy and 1 Gy in a dose-dependent manner (1.45 times higher for the 0.1 Gy IR only group compared to the control group vs. 1.90 times higher for the 1 Gy IR only group compared to the control group, *p* = 0.010). In contrast, serum HMGB1 was significantly lower in the IR + GL groups compared to the IR only groups for both 0.1 and 1 Gy (Fig. 2B).

**Figure 3:**
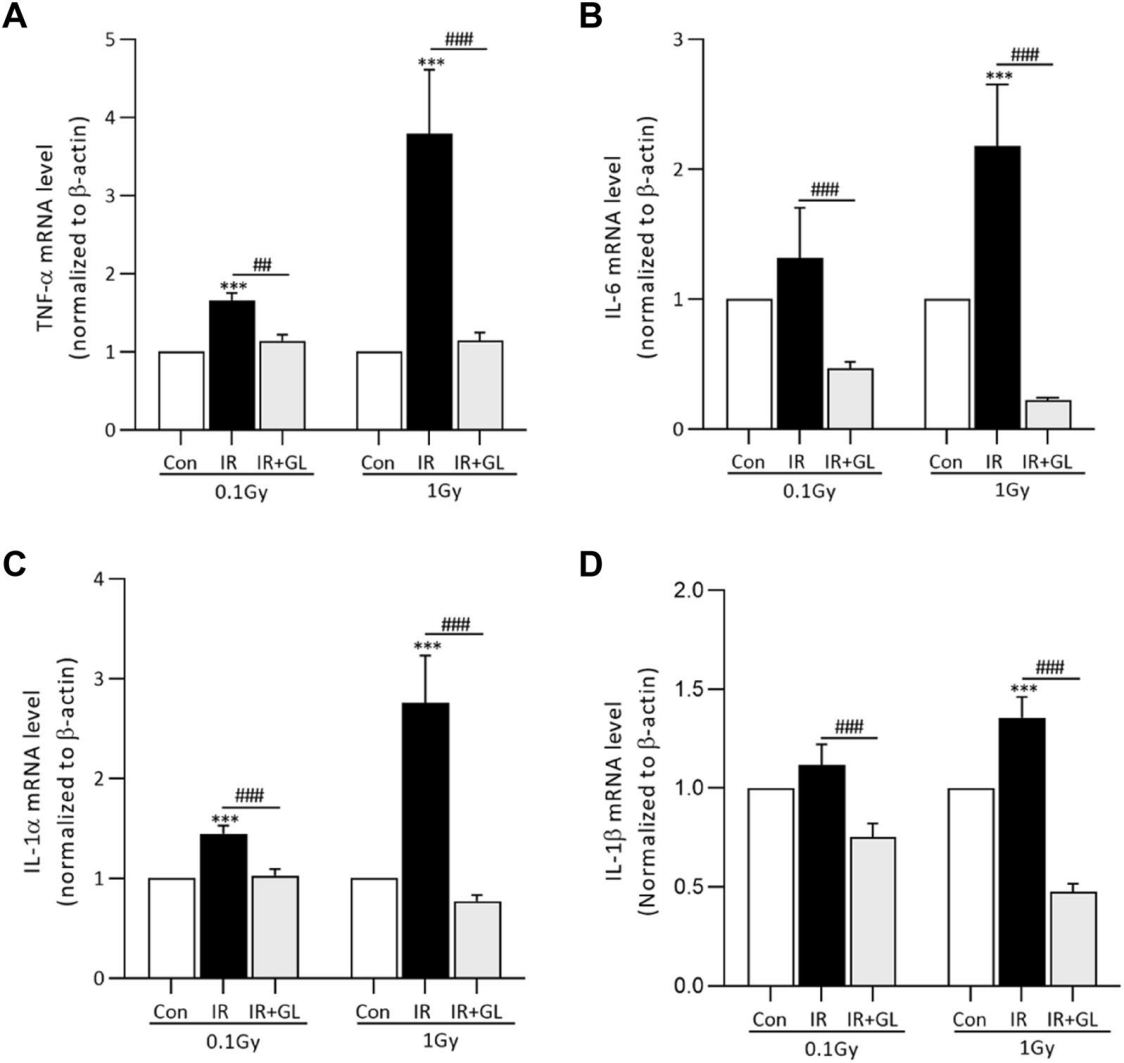
The mRNA expression of (**A**) TNF-α, (**B**) IL-6, (**C**) IL-1α, and (**D**) IL-1β according to radiation exposure. The mRNA expressions of pro-inflammatory cytokines were higher in the IR only groups compared to the controls with/without statistical significance, whereas it was significantly lower in the IR + GL groups than the IR only groups. Results are expressed as means ± SEM from duplicate experiments (n = 5) performed in each group. The asterisk (*) indicates significant differences between the control and IR groups (both IR only and IR + GL). The hash sign (#) indicates significant differences between the IR only groups and IR + GL groups. ***p < 0.001, ##*p* < 0.01, ###*p* < 0.001. *IR* irradiation, *GL* glycyrrhizin.

The mRNA expressions of pro-inflammatory cytokines including TNF-α, IL-6, IL-1α and IL-1β were significantly higher in the IR only groups compared to the control group, except for IL-6 and IL-1β in the 0.1 Gy IR group. The degree of elevation for TNF-α and IL-1α in the IR only groups compared to the control group was dose dependent: 3.80 times (TNF-α) and 2.75 times (IL-1α) in the 1 Gy IR only groups versus 1.65 times (TNF-α) and 1.44 times (IL-1α) in the 0.1 Gy IR only groups (*p* = 0.027 for TNF-α and *p* = 0.022 for IL-1α, respectively). In terms of IL-6 and IL-1 β, there was a tendency for mRNA expression to increase as the radiation dose increased, but without statistical significance (*p* = 0.176 for IL-6 and *p* = 0.125 for IL-1β). On the other hand, the mRNA expressions of all pro-inflammatory cytokines were significantly lower in the IR + GL groups than the IR only groups (*p* < 0.001, respectively) (Fig. 3A–D).

Furthermore, we investigated the protein expression of pro-inflammatory cytokines after radiation exposure. Protein expression was significantly higher in the IR only groups compared to the control group (0.1 Gy and 1 Gy; *p* = 0.008 for IL-1α, 0.1 Gy; *p* = 0.008 and 1 Gy; *p* = 0.047 for IL-1β), whereas it was mitigated in the IR + GL groups compared to the IR only groups (0.1 Gy and 1 Gy; *p* = 0.008 for IL-1α, 0.1 Gy and 1 Gy; *p* = 0.269, *p* = 0.730 for IL-1β). In the case of TNF-α and IL-6, protein expression increased after radiation exposure compared to the controls and decreased after pretreatment of glycyrrhizin, although without statistical significance (*p* > 0.05; except TNF-α at 1 Gy, *p* = 0.008) (Supplementary Fig. 2A–D).

### Preservation of cell viability in radiation-exposed isolated splenocytes with glycyrrhizin

**Figure 4:**
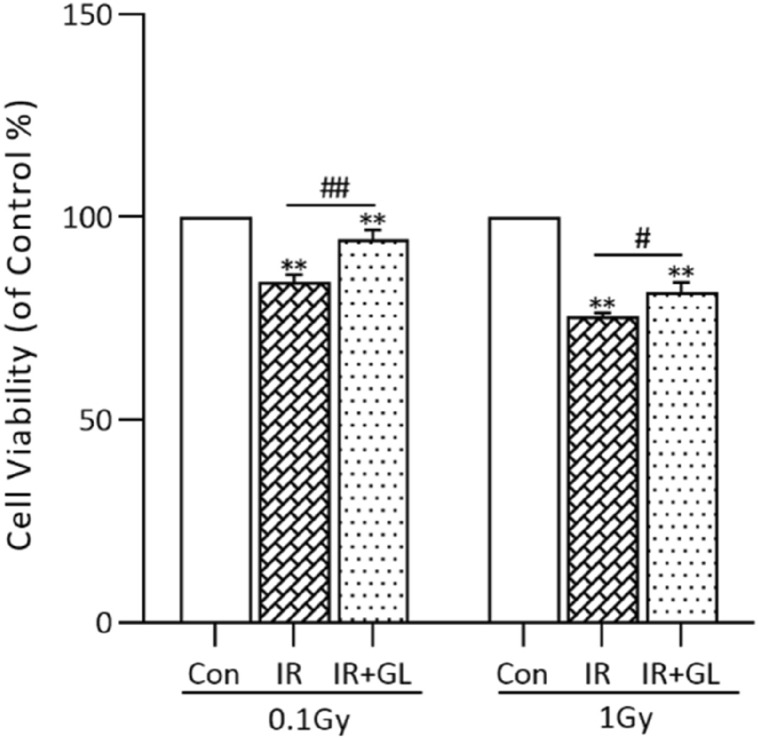
Cell viability of the isolated splenocytes according to radiation dose. Cell viabilities were significantly lower in the IR only groups (both in 0.1 Gy and 1 Gy) compared to the control group, whereas they were significantly higher in the IR + GL groups compared to the IR only groups. Results are expressed as means ± SEM from duplicate experiments for each group. The asterisk (*) indicates significant differences between the control and IR groups (both of the untreated and GL treated groups). The hash sign (#) indicates significant differences between the IR groups and IR + GL groups. **p < 0.01, ##*p* < 0.01, #*p* < 0.05. *IR* irradiation, *GL* glycyrrhizin.

Cell viability was significantly lower in the IR groups compared to the control group (*p* < 0.001, respectively), whereas it significantly improved in the IR + GL groups compared to the IR only groups (0.006 for 0.1 Gy and 0.049 for 1 Gy). In terms of radiation dose, the cell viability of the 0.1 Gy IR only group was significantly higher than the 1 Gy IR only group (*p* = 0.002) (Fig. 4).

### Expression of HMGB1 and pro-inflammatory cytokines after radiation exposure in isolated splenocytes

**Figure 5:**
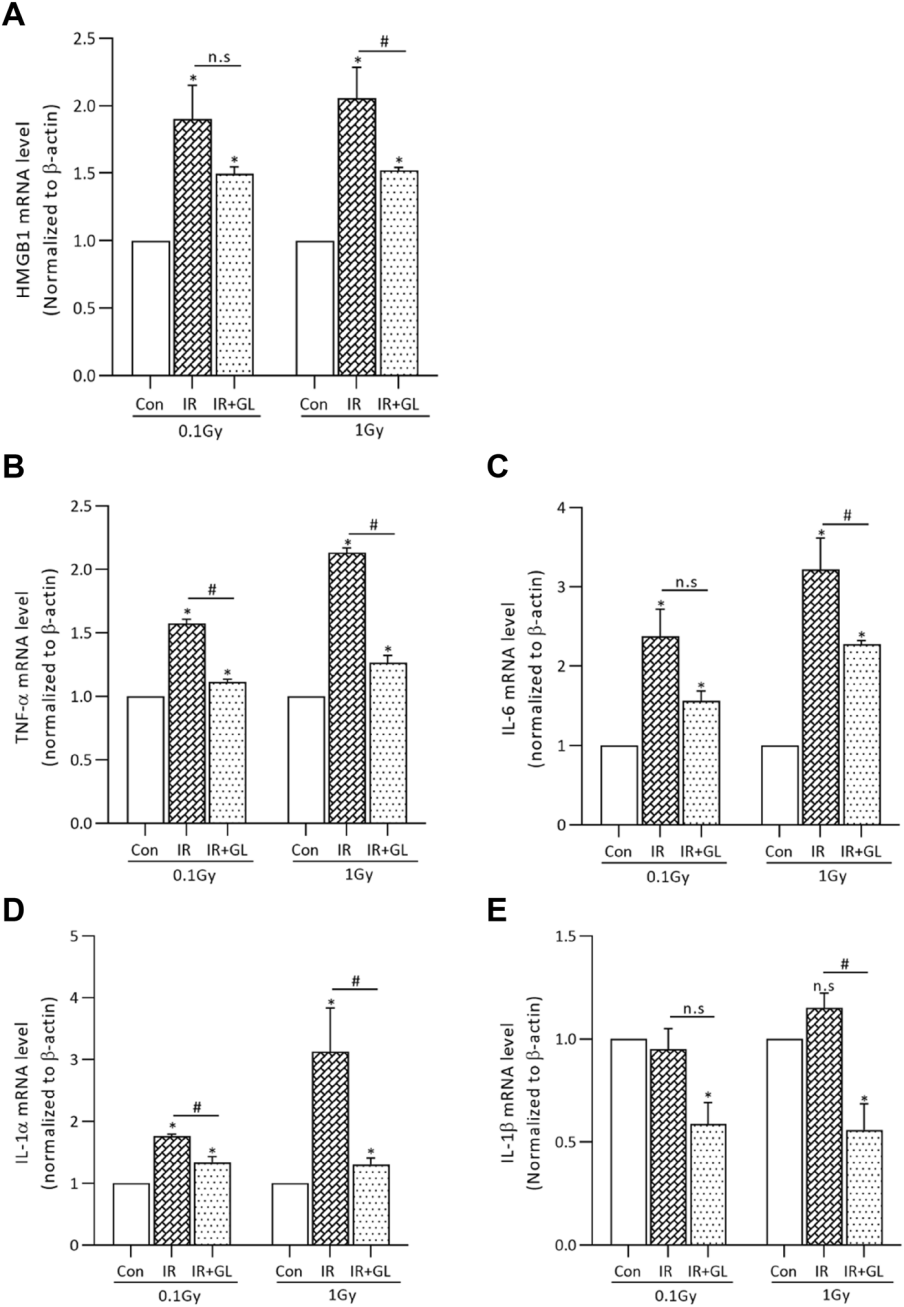
The mRNA expression of HMGB1 and pro-inflammatory cytokines in isolated splenocytes according to radiation dose. The mRNA expression levels of HMGB1 and pro-inflammatory cytokines were higher in the IR only group except for IL-1β (both in 0.1 Gy and 1 Gy) compared to the control group. With glycyrrhizin pretreatment, the mRNA expression levels of HMGB1 and pro-inflammatory cytokines were lower than IR only groups with and without statistical significance. Results are expressed as means ± SEM from duplicate experiments performed for each group. The asterisk (*) indicates statistically significant differences between the control and IR groups (both IR only and IR + GL). The hash sign (#) indicates significant differences between the IR only and IR + GL groups. *p < 0.05, #*p* < 0.05.

We also confirmed the protective effect of glycyrrhizin after radiation exposure in isolated splenocytes. The results for the isolated splenocytes demonstrated a similar tendency to those of radiation-exposed mice. The mRNA expression of HMGB1 and pro-inflammatory cytokines after radiation exposure were alleviated in the isolated splenocytes after glycyrrhizin treatment. The mRNA expression level of HMGB1 was significantly higher in the IR only groups compared to the control group (*p* = 0.028, both for 0.1 Gy and 1 Gy). In contrast, the mRNA expression of HMGB1 in the IR + GL groups was significantly lower compared to the IR only groups with and without statistical significance (*p* = 0.342 for 0.1 Gy, *p* = 0.028 for 1 Gy) (Fig. 5A).

In addition, the mRNA expression of pro-inflammatory cytokines including TNF-α, IL-6 and IL-1α were significantly higher in the IR only groups than the control group. The degree of increase in TNF-α, IL-6 and IL-1α in the IR only groups compared to the control group was dose dependent: 1.57 times (TNF-α), 2.38 times (IL-6) and 1.76 times (IL-1α) in the 0.1 Gy IR only groups versus 2.13 times (TNF-α), 3.22 times (IL-6) and 3.13 times (IL-1α) in the 1.0 Gy IR only groups. With glycyrrhizin pretreatment, the mRNA levels of the pro-inflammatory cytokines were lower than those of the IR only groups (Fig. 5B–E).

## Discussion

When pathogens such as microorganisms are recognized by specific receptors as pathogen-associated molecular patterns (PAMPs), a series of immune reactions occur in response to tissue damage and cellular death^24^. However, trauma or burns can also cause tissue damage, resulting in an immune response triggered without pathogens. In contrast to PAMPs, endogeneous molecules which incur immune response are defined as alarmin (or damage associated molecular patterns, DAMPs) and HMGB1 is an example of this category^24,25^. Radiation is one stimuli that cause tissue damage without pathogens^24^, and extracellular HMGB1 has been found to increase after irradiation^22,23^. In this study, we demonstrated that both mRNA expression and serum levels of HMGB1 and pro-inflammatory cytokines such as TNF-α, IL-6, IL-1α and IL-1β increase dose dependently after 0.1 Gy and 1 Gy radiation exposure. Furthermore, an increase in oxidative stress and a decrease in cell viability was observed after low-dose radiation exposure as well. Therefore, low-dose radiation exposure does not only act as an alarmin but also mediates oxidative stress, similar to what was observed in previous studies conducted on relatively high-dose radiation exposures^26^. Subsequently, HMGB1 levels can be lowered and oxidative stress reduced with GL, a direct inhibitor of HMGB1, that subsequently might allow the preservation of cell viability. Thus, HMGB1 could be a target for reducing sterile inflammatory response even after low-dose radiation exposure.

Radiation exposure is known as an initiator of sterile inflammation and can increase serum HMGB1 levels by inducing the active or passive release of HMGB1 from cells^17^. Although the cause of this release of HMGB1 is still not fully eluciated, inflammasomes such as node-like receptor protein 3 (NLRP3) are thought to play a role^25^. In addition, serum HMGB1 levels have been shown to increase with increasing radiation exposure^17^. These past findings was comparable with our results in that both mRNA expression and serum levels of HMGB1 increased after low-dose radiation exposure in a dose-dependent manner. As for extracelular HMGB1, it recruits leucocytes by binding to CXCL12 and mediates the production of cytokines by activating immune cells via TLR4, RAGE, TRL2 and TRL9 depending on its redox state^17,27^. In our study, pro-inflammatory cytokines including TNF-α and IL-1α significantly increased after radiation exposure in a dose-dependent manner. Whereas IL-1β and IL-6 increased in a dose-dependent manner, there was no significant difference between the control group and 0.1 Gy IR only group. According to a previous study, IL-1β and IL-6 also significantly increased both 2 weeks and 4 weeks after mice were exposed to 20 Gy dose of radiation^22^ and the reason for this discrepancy might be the lack of linear dose dependency between radiation dose and cytokine production, the time delay between radiation exposure and measurement, and differences in the experimental animal strains^28^ used in the previous study and ours.

GL inhibits the chemotactic and mitogenic activities of HMGB1 by directly binding to two DNA binding sites of HMGB1 (A and B box)^19,27^. In previous studies, GL could reduce the serum levels of inflammatory cytokines and HMGB1 after radiation exposure, resulting in ameliolating radiation-induced enteritis and radiation-induced lung injury^22,23^. This protective effect is also seen in our results with the pretreatment of GL attenuating both the mRNA and serum HMGB1 levels and significantly decreasing the level of pro-inflammatory cytokines even after low-dose radiation exposure that was approximately 0.5–15.4% of the radiation administered in previous studies. Furthermore, cell viability also significantly improved with GL, suggesting that GL has protective effects even after low-dose radiation exposure.

According to previous studies, low LET radiation exposure can cause DNA damage by oxidative stress which is mediated through the radiolysis of water rather than direct tissue injury or breakdown of DNA^29,30^. By radiolysis, free radicals such as the superoxide anion, hydroxyl radicals and hydrogen perioxides are generated which are toxic to adjacent cells^29^. Similar to previous studies, oxidative stress increased and cell viability decreased compared to the control group with radiation, even when the radiation dose was small at 0.1 Gy. But, the level of oxidative stress did not significantly differ between 0.1 Gy and 1 Gy. This might be because the relationship between radiation dose and oxidative stress is not linear, possibly due to antioxidant mechanisms such as the upregulation of GSH synthesis^31^. that do not attenuate excessive oxidative stress in a dose-dependent manner. Conversely, if oxidative stress can be sufficiently countered by antioxidant mechanisms or methods that boost preexisting antioxidant mechanisms in the body, the harmful effects of low-dose radiation exposure from diagnostic imaging can be prevented or markedly ameliorated. However, the relationship between exposured dose and oxidative stress and the exact mechanism behind this relationship needs to be investigated in a future study.

There are several limitations to our study. First, as is common in many animal studies, the results of this study can only be considered potential indicators of what might occur in humans, and are not confirmative of the actual complex clinical situations. Second, the number of animals used in the experiment was relatively small. Third, downstream receptors of HMGB1 such as TLR or RAGE were not investigated. Finally, we did not evaluate DNA damage such as double strand breakdown that might occur after radiation exposure.

In conclusion, even low-dose radiation can induce sterile inflammation by increasing serum HMGB1 and pro-inflammatory cytokines but GL can potentially ameliorate this sterile inflammatory process by inhibiting HMGB1, and thereby preserve cell viability.

## Material and methods

### Animal preparation and whole-body radiation exposure

**Figure 6:**
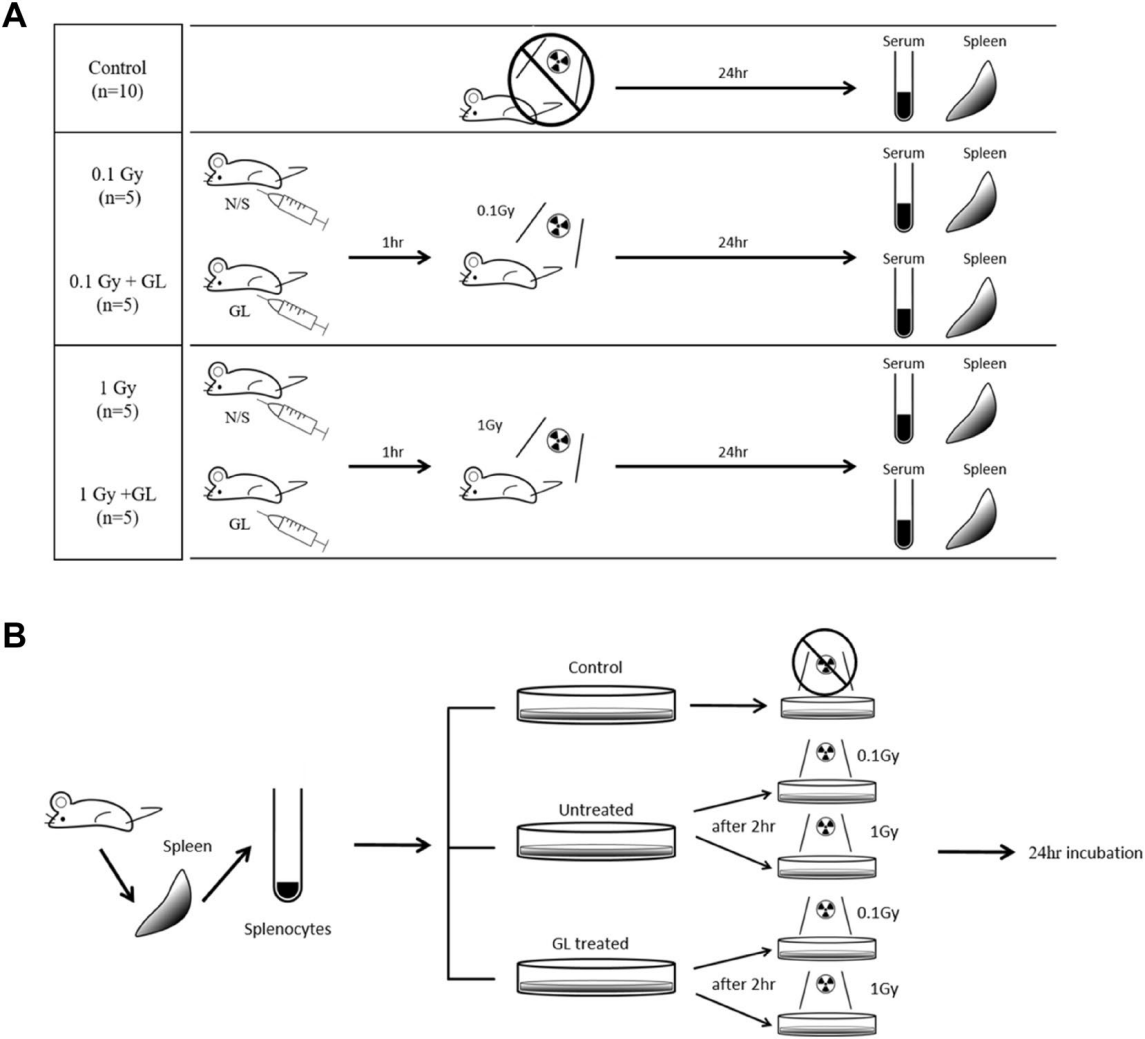
Scheme of the animal experiments. (**A**) Thirty mice were divided into 5 groups which were the control (n = 10), 0.1 Gy IR only (n = 5), 0.1 Gy IR + GL (n = 5), 1 Gy IR only (n = 5) and 1 Gy IR + GL (n = 5) group. Except for the control group, all mice received whole body irradiation with a total single dose of 0.1 Gy or 1 Gy (300 kV, 12.5 mA). GL was administrated intraperitoneally before 2 h of irradiation. All mice were sacrificed after 24 h of radiation exposure and blood samples were collected and spleen tissue was harvested. (**B**) To assess cell viability and mRNA expression , the radiation-exposed splenocytes were incubated for 24 h after being isolated from the mice (no-irradiation). These samples were divided into the five groups (Control, 0.1 Gy IR only, 0.1 Gy IR + GL, 1 Gy IR only, and 1 Gy IR + GL) as well. GL was applied 2 h before radiation exposure for the 0.1 Gy IR + GL and 1 Gy IR + GL groups. *IR* irradiation, *GL* glycyrrhizin, *N/S* normal saline.

All experimental procedures were approved by the Department of Laboratory Animal Resonances, Yonsei University and the Animal Ethics Committee with the ID number of 2017–0271 and were performed in accordance with IACUC guidelines and ARRIVE guidelines. A total of 30 BALB/c male mice about 5 weeks old and weighing 20–22 g were chosen for this experiment. All animals were kept in plastic cages and fed in a sterile 12 h light and 12 h dark cycle with 50 ± 10% humidity and 22 ± 2 °C temperature during the experiment. After a 1-week acclimatization period, the mice were divided into 5 groups: control (n = 10), 0.1 irradiation (IR) only (n = 5), 0.1 Gy IR + GL (n = 5), 1 Gy IR only (n = 5) and 1 Gy IR + GL (n = 5) (Fig. 6A). GL (Selleck Chemicals, Houston, TX) was dissolved in 2% dimethyl sulfoxide (DMSO), 30% polyethylene glycol and 2% polyoxyethylene sorbitan monooleate, which was diluted with saline and administrated intraperitoneally (100 mg/kg) 2 h before radiation exposure in both the IR + GL groups. The same dose of normal saline was applied to the control group and all ‘IR only’ groups intraperitoneally. Except for the control group, all mice were exposed to whole body radiation with a total single dose of 0.1 Gy or 1 Gy (300 kV, 12.5 mA) using an irradiator, X-Rad320 (Precision Inc., North Branford, CT) according to their group classification, respectively. The mice were sacrificed 24 h after radiation exposure and blood samples were collected and spleen tissue was harvested.

### Splenocyte isolation

The spleen of each mice was aseptically removed using scissors and forceps and placed in a sterile petri dish containing the RPMI1640 medium with 5% fetal bovine serum. Single-cell suspensions were prepared as follows. The mice spleens were gently crushed between the glass microscope slides. The crushed tissue was washed with the RPIM 1640 medium to collect cells. Debris was removed by filtration through a 40 μM nylon cell strainer and the passed cell suspension was then centrifuged at 12,000 rpm for 20 min. Then, pellets were lysated using ACK (ammonium-chloride-potassium) lysis buffer for 10 min to lyse the red blood cells (RBC). After centrifuging at 12,000 rpm for 5 min, pellets were newly collected and washed twice with 1 × phosphate buffered saline (1 × PBS) and centrifuged at 12,000 rpm for 5 min.

### Serum collection

Blood samples were collected without anticoagulants from the mice by a cardiac puncture through the right atrium with a 26-gauge syringe needle. The blood was placed into a 1.5-ml tube and centrifuged at 13,000 rpm for 20 min at room temperature. The serum samples were used for the enzyme-linked immunosorbent assay (ELISA) and malondialdehyde (MDA) assay.

### Oxidative stress quantification using MDA

Oxidative stress was determined by measuring the formation of MDA, which is a product of membrane lipid peroxidation using 2-ThioBarbituric Acid Reactive Substances. The MDA level was calculated as MDA equivalents using a commercial kit (Cat #10009055, Cayman Chemical, Ann Arbor, MI) according to the manufacture’s instructions.

### Flow cytometry analysis

To measure cellular reactive oxygen species (ROS), the collected splenocyte cells were stained with 1 μM chloromethyl-dichlorodihydrofluorescein diacetate, acetyl ester (CM-H_2_DCFDA, Invitrogen, Carlsbad, CA) for 20 min in a 37 °C incubator. The stained cells were analyzed using BD LSRII flow cytometry with BD FACSDiva software (BD Bioscience, San Jose, CA).

### Enzyme-linked immunosorbent assay (ELISA)

To measure mouse serum HMGB1, IL-1α, IL-1β, IL-6, TNF-α and oxidative 8-OHdG DNA damage we used the mouse ELISA kit (Cat#SEKM-0145, Solarbio Science & Technology, Beijing, China), IL-1α (ab199076, Abcam, Cambridge, MA), IL-1β (ab197742, Abcam, Cambridge, MA), IL-6 (ab222503, Abcam, Cambridge, MA), TNF-α (ab208348, Abcam, Cambridge, MA), and oxdative 8-OHdG DNA damage (8-OHdG Quantitation) (STA-210-T, Cell biolabs, San Diego, CA) according to each manufacturer’s instructions. Absorbance levels of concentrations were measured at 450 nm using a microplate reader (Molecular Devices, Sunnyvale, CA).

### RNA extraction and quantitative real-time polymerase chain reaction

Total RNA was obtained from the isolated splenocytes using a commercial kit (Hybrid-R kit 305-101, GeneAll Biotechnology, Seoul, Korea) and 1 µg of total RNA was reverse transcribed with amfiRivert cDNA synthesis (GenDEPOT, Houston, TX) according to the manufacturer’s instructions. The mRNA expression levels of HMGB1, IL-1α, IL-β, IL-6 and TNF-α were assessed by real-time qRT-PCR using the SYBR-Green reagent (GenDEPOT, Houston, TX) with the ABI7500 real-time PCR system (Applied biosystems, Foster city, CA). Gene expression was relatively quantified using β-actin as the internal reference. All real-time PCR reactions were conducted in duplications and threshold (Ct) values were analyzed by the 2^−ΔΔCt^ method. The primer sequences for real-time PCR are shown in Table 1.

**Table 1:** Primer sequence for Real-time PCR.

Gene	Forward sequence (5′-3′)	Reverse sequence (5′-3′)
β-actin	CATTGCTGACAGGATGCAGAAGG	TGCTGGAAGGTGGACAGTGAGG
HMGB1	CCAAGAAGTGCTCAGAGAGGTG	GTCCTTGAACTTCTTTTTGGTCTC
TNF-α	GGTGCCTATGTCTCAGCCTCTT	GCCATAGAACTGATGAGAGGGAG
IL-1α	ACGGCTGAGTTTCAGTGAGACC	CACTCTGGTAGGTGTAAGGTGC
IL-1β	GCAACTGTTCCTGAACTCAACT	ATCTTTTGGGGTCCGTCCAACT
IL-6	TACCACTTCACAAGTCGGAGGC	CTGCAAGTGCATCATCGTTGTTC

### Cell viability

Isolated splenocytes were spilt into 96-well plates (1 × 10^6^ cells/well) and incubated at 37 °C in humidified 5% CO_2_ for 24 h (Fig. 6B). Cells were divided into five groups: control, 0.1 Gy IR only, 0.1 Gy IR + GL, 1 Gy IR only, and 1 Gy IR + GL. The incubated cells were treated with GL 2 h before radiation exposure. Except for the control group, all 96-well plates received irradiation with a total single dose of 0.1 Gy or 1 Gy (300 kV, 12.5 mA) using an irradiator, X-Rad320 (Precision Inc., North Branford, CT). After 24 h of radiation exposure, the cells were assessed with the CCK-8 assay kit (Dojindo Laboratories, Kumamoto, Japan).

### Immunofluorescence staining

The isolated splenocyte cells were fixed in 4% paraformaldehyde for 20 min and rinsed twice with PBS (Phosphated Buffered Saline). The cells were permeabilized with 0.2% Triton X-100 for 25 min and then blocked with 3% BSA for 30 min. Cells were incubated overnight at 4 °C with a primary antibody against γ-H2A.X (ser139) (1:250, #2557, Cell signaling, MA, USA). The next day, the cells were washed twice with PBS and incubated with a secondary antibody against Alexa Fluor 594 goat anti-rabbit IgG (H + L) (1:500, Invitrogen, MA, USA) in the dark at room temperature for 1 h. The cells were then washed twice with PBS and diluted with DAPI. The stained slides were visualized with confocal microscopy (LSM700, Carl Zeiss GmbH, Jena, Germany).

### Statistical analysis

All experiments were independently repeated at least twice. Statistical analyses were performed using Prism 9.0.0 (Graphpad, San Diego, CA) and SPSS v25.0 (IBM Corp., New York, NY). To determine the statistical significance of differences, the non-parametric Mann–Whitney U test was used to compare two groups at a time, and the Kruskal–Wallis test was performed with post-hoc analysis (Dunn’s test) to compare three groups. *p* < 0.05 was considered to indicate statistical significance.

## Supplementary Information

Supplementary Information.

## References

[1] Hall EJ, Giaccia AJ (2019). Radiobiology for the radiologist.

[2] Vaiserman A, Koliada A, Zabuga O, Socol Y (2018). Health impacts of low-dose ionizing radiation: Current scientific debates and regulatory issues. Dose Response.

[3] Mullenders L, Atkinson M, Paretzke H, Sabatier L, Bouffler S (2009). Assessing cancer risks of low-dose radiation. Nat. Rev. Cancer.

[4] Council NR, Studies DEL, Research BRE, Radiation CAHRELLI. *Health Risks from Exposure to Low Levels of Ionizing Radiation: BEIR VII Phase 2* (National Academies Press, 2006). 25077203

[5] Hong JY, Han K, Jung JH, Kim JS (2019). Association of exposure to diagnostic low-dose ionizing radiation with risk of cancer among youths in South Korea. JAMA Netw. Open.

[6] Haylock RGE, Gillies M, Hunter N, Zhang W, Phillipson M (2018). Cancer mortality and incidence following external occupational radiation exposure: An update of the 3rd analysis of the UK national registry for radiation workers. Br. J. Cancer..

[7] Andersson U, Tracey KJ (2011). HMGB1 is a therapeutic target for sterile inflammation and infection. Annu. Rev. Immunol..

[8] Chen GY, Nunez G (2010). Sterile inflammation: Sensing and reacting to damage. Nat. Rev. Immunol..

[9] Wang H, Bloom O, Zhang M (1999). HMG-1 as a late mediator of endotoxin lethality in mice. Science.

[10] Urbonaviciute V, Furnrohr BG, Meister S (2008). Induction of inflammatory and immune responses by HMGB1-nucleosome complexes: Implications for the pathogenesis of SLE. J. Exp. Med..

[11] Harris HE, Andersson U, Pisetsky DS (2012). HMGB1: A multifunctional alarmin driving autoimmune and inflammatory disease. Nat. Rev. Rheumatol..

[12] Yang D, Chen Q, Yang H, Tracey KJ, Bustin M, Oppenheim JJ (2007). High mobility group box-1 protein induces the migration and activation of human dendritic cells and acts as an alarmin. J. Leukoc. Biol..

[13] Andersson U, Wang H, Palmblad K (2000). High mobility group 1 protein (HMG-1) stimulates proinflammatory cytokine synthesis in human monocytes. J. Exp. Med..

[14] Scaffidi P, Misteli T, Bianchi ME (2002). Release of chromatin protein HMGB1 by necrotic cells triggers inflammation. Nature.

[15] Hori O, Brett J, Slattery T (1995). The receptor for advanced glycation end products (RAGE) is a cellular binding site for amphoterin. Mediation of neurite outgrowth and co-expression of rage and amphoterin in the developing nervous system. J. Biol. Chem..

[16] Park JS, Svetkauskaite D, He Q (2004). Involvement of toll-like receptors 2 and 4 in cellular activation by high mobility group box 1 protein. J. Biol. Chem..

[17] Wang L, He L, Bao G, He X, Fan S, Wang H (2016). Ionizing radiation induces HMGB1 cytoplasmic translocation and extracellular release. Guo Ji Fang She Yi Xue He Yi Xue Za Zhi..

[18] Zhou H, Jin C, Cui L (2018). HMGB1 contributes to the irradiation-induced endothelial barrier injury through receptor for advanced glycation endproducts (RAGE). J. Cell Physiol..

[19] Mollica L, De Marchis F, Spitaleri A (2007). Glycyrrhizin binds to high-mobility group box 1 protein and inhibits its cytokine activities. Chem. Biol..

[20] Asl MN, Hosseinzadeh H (2008). Review of pharmacological effects of Glycyrrhiza sp. and its bioactive compounds. Phytother. Res..

[21] Gong G, Xiang L, Yuan L (2014). Protective effect of glycyrrhizin, a direct HMGB1 inhibitor, on focal cerebral ischemia/reperfusion-induced inflammation, oxidative stress, and apoptosis in rats. PLoS ONE.

[22] Zheng L, Zhu Q, Xu C (2020). Glycyrrhizin mitigates radiation-induced acute lung injury by inhibiting the HMGB1/TLR4 signalling pathway. J. Cell Mol. Med..

[23] Zhang XM, Hu X, Ou JY (2020). Glycyrrhizin ameliorates radiation enteritis in mice accompanied by the regulation of the HMGB1/TLR4 pathway. Evid Based Complem. Altern. Med..

[24] Bianchi ME (2007). DAMPs, PAMPs and alarmins: All we need to know about danger. J. Leukoc. Biol..

[25] Vénéreau E, Ceriotti C, Bianchi ME (2015). DAMPs from cell death to new life. Front. Immunol..

[26] Liu L, Qu H, Qin H (2019). NOD2 agonist murabutide alleviates radiation-induced injury through DNA damage response pathway mediated by ATR. J. Cell Physiol..

[27] Andersson U, Rauvala H (2011). Introduction: HMGB1 in inflammation and innate immunity. J. Intern. Med..

[28] Schaue D, Kachikwu EL, McBride WH (2012). Cytokines in radiobiological responses: A review. Radiat. Res..

[29] Kim W, Lee S, Seo D (2019). Cellular stress responses in radiotherapy. Cells.

[30] Nuszkiewicz J, Woźniak A, Szewczyk-Golec K (2020). Ionizing radiation as a source of oxidative stress—The protective role of melatonin and vitamin D. Int. J. Mol. Sci..

[31] Azzam EI, Jay-Gerin JP, Pain D (2012). Ionizing radiation-induced metabolic oxidative stress and prolonged cell injury. Cancer Lett..

