# Supplementary material for: Glycyrrhizin ameliorating sterile inflammation induced by low-dose radiation exposure
Source: Sci Rep. 2021 Sep 15;11:18356. doi: 10.1038/s41598-021-97800-8 (PMC8443578; doi:10.1038/s41598-021-97800-8)
Supplement: Supplementary file 1 — Supplementary Information. [file 41598_2021_97800_MOESM1_ESM.docx]

Supplementary information for :

**Glycyrrhizin ameliorating sterile inflammation induced by Low-dose Radiation Exposure**

Hyung Cheol Kim^1*^, Hyewon Oh^1*^, Je Sung You^2^, Yong Eun Chung^1^

^1^Department of Radiology, Yonsei University College of Medicine, Seoul, Republic of Korea

^2^Department of Emergency Medicine, Yonsei University College of Medicine, Seoul, Republic of Korea

*Both authors contributed equally to this work

Correspondence:

Yong Eun Chung, MD, PhD

Department of Radiology, Severance Hospital, Yonsei University College of Medicine, 50-1 Yonsei-ro, Seodaemun-gu, Seoul 03722, Republic of Korea, Tel: 82-2-2228-7400, Fax: 82-2-2227-8337, E-mail: [yelv@yuhs.ac](mailto:yelv@yuhs.ac)

Supplementary Figure 1.


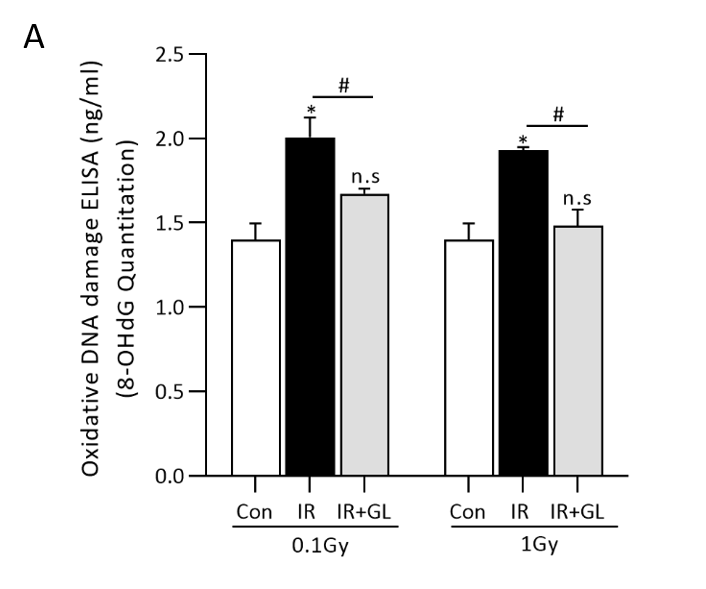


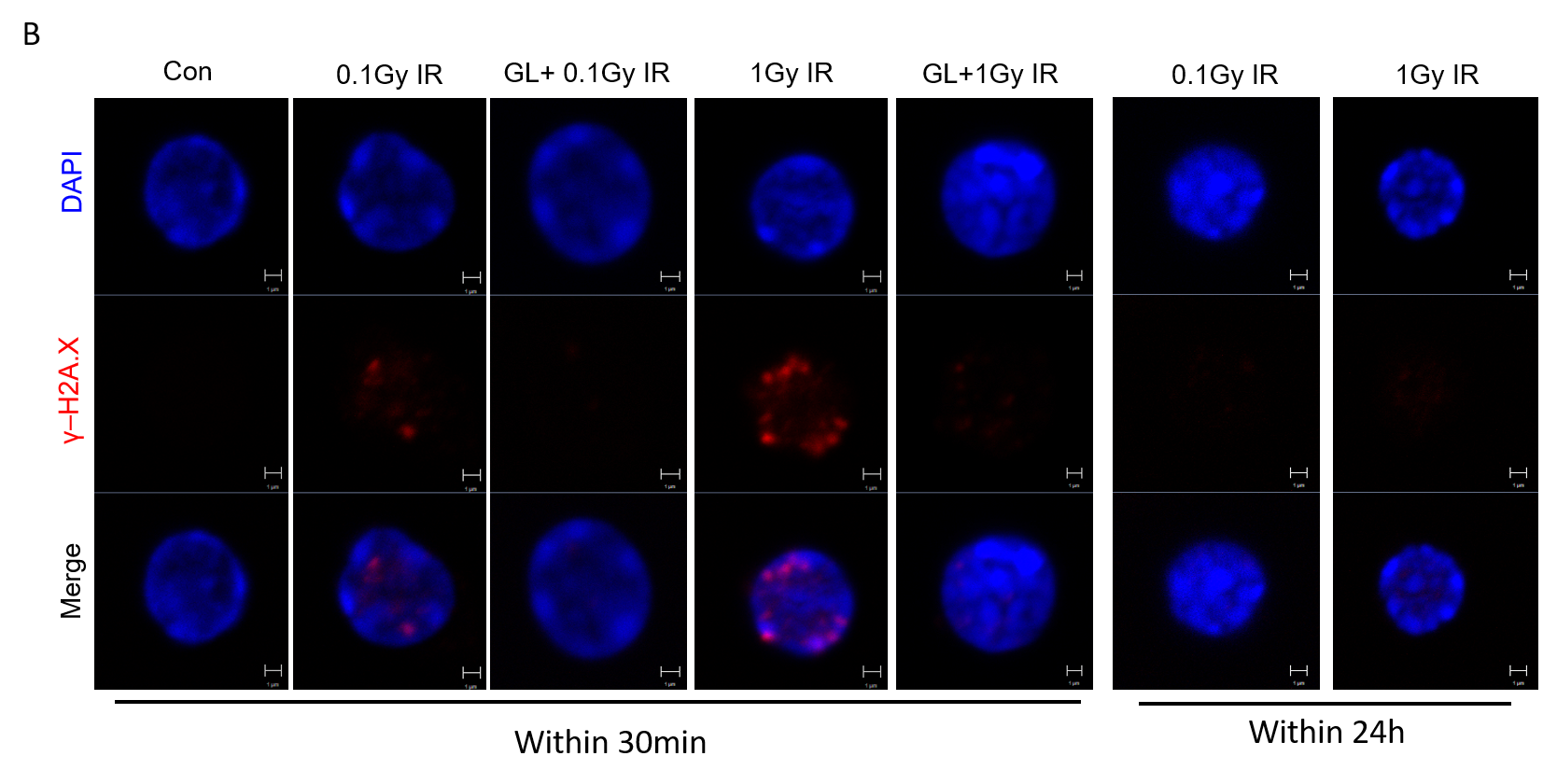


**Supplementary Figure 1. Expression of DNA-damage related markers by oxidative stress.** (A) The graph compared the oxidative DNA damage ELISA results for the IR with/ without GL groups compared to the control group. Oxidative DNA damage was significantly higher in the IR only groups compared to the controls, whereas it was lower in the GL+IR groups. (B) The immunofluorescence of γ-H2A.X showed foci formation in the IR only groups (in both 0.1 Gy and 1 Gy) within 30min, whereas foci formation disappeared within 24h. Foci formation was mitigated in the IR+ GL groups. Magnification=40X, Scale bar =10μm. Results are expressed as means ± SEM from duplicate experiments (n=4) performed in each group. The asterisk (*) indicates significant differences between the control and IR groups (both IR and IR+GL). The hash sign (#) indicates significant differences between the IR only groups and IR+GL groups. *P<0.05 and #P<0.05. Abbreviation: IR; Irradiation, GL; Glycyrrhizin, ELISA; Enzyme-Linked Immunosorbent Assay.

Supplementary Figure 2.


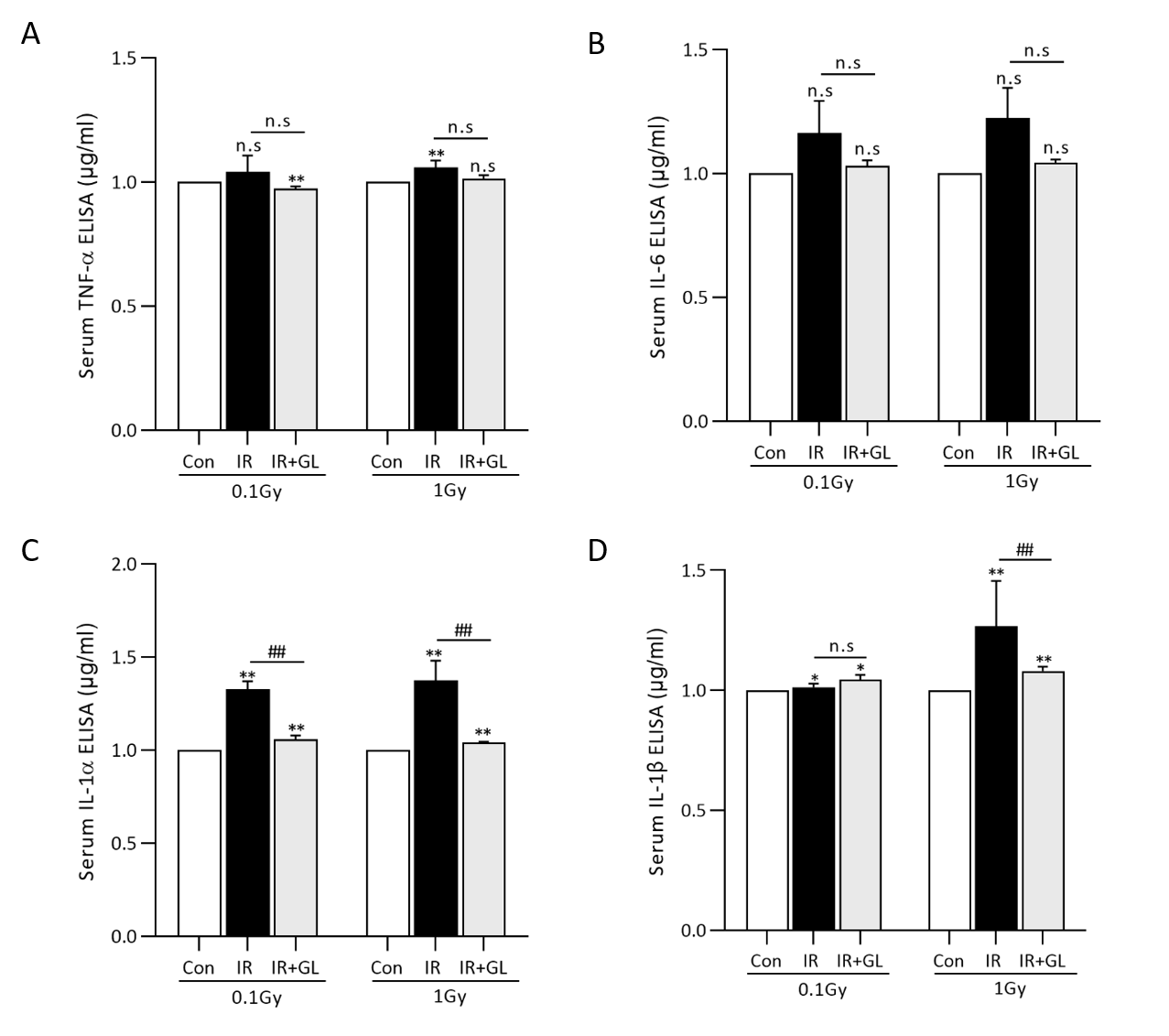


**Supplementary Figure 2.** The protein expression of (A) TNF-α, (B) IL-6, (C) IL-1α and (D) IL-1β according to radiation dose using ELISA. The protein expressions of pro-inflammatory cytokines were higher in the IR only groups compared to control group, whereas they were lower in the IR+GL groups compared to the IR only groups except IL-1β after exposure to 0.1 Gy, with or without statistical significance. Results are expressed as means ± SEM from duplicate experiments (n=5) performed in each group. The asterisk (*) indicates significant differences between the control and IR groups (both IR and IR+GL). The hash sign (#) indicates significant differences between the IR only groups and IR+GL groups. *P<0.05, **P<0.01 and ##P<0.01. Abbreviation: IR; Irradiation, GL; Glycyrrhizin, ELISA; Enzyme-Linked Immunosorbent Assay.
